# The Epidemiology of Major Trauma During the First Wave of COVID-19 Movement Restriction Policies: A Systematic Review and Meta-analysis of Observational Studies

**DOI:** 10.1007/s00268-022-06625-7

**Published:** 2022-06-20

**Authors:** Marcello Antonini, Madeleine Hinwood, Francesco Paolucci, Zsolt J. Balogh

**Affiliations:** 1 School of Medicine and Public Health, University of Newcastle, University Dr, Callaghan, NSW 2308 Australia; 2 Medical Research Institute, Lookout Road, New Lambton Heights, NSW 2305 Australia; 3 Newcastle Business School, University of Newcastle, Hunter St &, Auckland St, Newcastle, NSW 2300 Australia; 4 Department of Sociology and Business Law, University of Bologna, Strada Maggiore 45, 40126 Bologna, Italy; 5 Division of Surgery, John Hunter Hospital, Locked Bag No. 1, Hunter Region Mail Centre, Newcastle, NSW 2310 Australia

## Abstract

**Background:**

The objective of this systematic review is to investigate changes in the epidemiology of major trauma presentations during the implementation of movement restriction measures to manage the first wave of the SARS-CoV-2 (COVID-19) pandemic.

**Methods:**

A systematic search in six databases, as well as a search of grey literature was performed from January 2020 to August 2021. Estimates were pooled using random-effects meta-analysis. The certainty of evidence was rated according to the GRADE approach. The review is reported using both PRISMA guideline and the MOOSE checklist.

**Results:**

In total, 35 studies involving 36,987 patients were included. The number of major trauma admissions overall decreased during social movement restrictions (−24%; *p* < 0.01; 95% CI [−0.31; −0.17]). A pooled analysis reported no evidence of a change in the severity of trauma admissions (OR:1.17; 95%CI [0.77, 1.79], *I*^2^ = 77%). There was no evidence for a change in mortality during the COVID-19 period (OR:0.94, 95%CI [0.80,1.11], *I*^2^ = 53%). There was a statistically significant reduction in motor vehicle trauma (OR:0.70; 95%CI [0.61, 0.81], *I*^2^ = 91%) and a statistically significant increase in admissions due to firearms and gunshot wounds (OR:1.34; 95%CI [1.11, 1.61], *I*^2^ = 73%) and suicide attempts and self-harm (OR:1.41; 95%CI [1.05, 1.89], *I*^2^ = 39%).

**Conclusions and relevance:**

Although evidence continues to emerge, this systematic review reports some decrease in absolute major trauma volume with unchanged severity and mortality during the first wave of COVID-19 movement restriction policies. Current evidence does not support the reallocation of highly specialised trauma professionals and trauma resources.

*Registration* PROSPERO ID CRD42020224827.

**Supplementary Information:**

The online version contains supplementary material available at 10.1007/s00268-022-06625-7.

## Introduction

The current SARS-CoV-2 (COVID-19) pandemic has posed unprecedented challenges for the organisation of healthcare systems and their financing. The rapid increase in hospitalisations required the suspension of elective medical services across a number of jurisdictions and reduced resources to hospital units that compete for the same resources as those used to treat COVID-19 [1]. In addition, resources including equipment and medical personnel were diverted in anticipation of managing outbreaks [2]. Trauma care and all its components were one of the most affected by the rearrangement of health care services during the first months of the pandemic [2, 3]. The justification for diverting resources and personnel from trauma units was driven by expectations that the contextual policies implemented to stop the spread of the virus such as lockdown policies, stay at home orders (SAH), and suspension of social activities (i.e. sports, entertainment, closure of pubs and restaurants, etc.) [4–6] would significantly reduce major trauma presentations. Nevertheless, researchers and clinicians have expressed concerns regarding the negative impact of COVID-19 outbreaks on the ability of health care systems to provide timely assessment and acute therapies to patients with major trauma [7, 8].

In this systematic review and meta-analysis, we aimed to investigate changes in the epidemiology of major trauma presentations during the implementation of movement restriction measures (such as lockdowns) to manage the first wave of the COVID-19 pandemic. This setting provides a relatively more homogeneous setting for a cross-country analysis, as subsequent waves were likely to be more heterogenous due to country-specific determinants such as additional investments in hospital resources, the impact of the first wave, vaccine availability.

We hypothesised that: (a) social restrictions will lead to a reduction in major trauma admissions; (b) there will be an increase in the severity of major trauma presentations during periods of social restriction, compared with pre-pandemic; and (c) there will be a reduction in traffic-related injuries and a corresponding increase in trauma at home.

## Methods

### Search strategy

The protocol for this review is available online (PROSPERO; CRD42020224827). This systematic review and meta-analysis aligns with the PRISMA guidelines [9]. The systematic search was initially performed in six databases on 19 January 2021, MEDLINE, Embase, CINAHL, the Cochrane Library, the WHO COVID-19 and LitCovid. A search of grey literature was also conducted via Google in the same timeframe, adopting the same search strategy used in Medline. We defined terms for SARS-CoV-2, policy restrictions, trauma, and hospitalisations/caseloads based on cohort studies reporting on major trauma presentations both before and after the COVID-19 pandemic outbreak [7, 10–12], and via review of governmental documents and media sources. The search strategy was created by the authors and peer-reviewed by a senior librarian. The search was updated on 26 July 2021 prior to submission. The full search strategy is available in the supplementary Appendix (Table A1–A5).

### Eligibility criteria

Major trauma was defined as per patients requiring trauma resuscitation based on institutional criteria on arrival to the emergency department. Although this definition may not be uniform across studies like an Injury Severity Score (ISS) based inclusion criteria, it allows consistency for comparison of pre-pandemic and pandemic volumes by using the same criteria in individual institutions.

Cohort studies were included if they reported differences on the number of admissions, between patients admitted due to major trauma after the COVID-19 pandemic outbreak and patients admitted due to major trauma before the COVID-19 pandemic outbreak in the respective health care settings (see Table A6 in the supplementary material). Also articles not published in English language and commentaries or editorials were excluded.

### Study selection and data extraction

We performed de-duplication in EndNote [13] and all records were exported to Covidence [14] for screening. Two reviewers (MA, MH) independently screened the titles and abstracts for relevance, and then extracted and selected relevant full-text records. Discrepancies were resolved through discussion at each stage, with any disputes of eligibility resolved by a third author where required (ZJB).

Two authors (MA and MH) developed the data extraction form in Excel. The form records bibliographic information, the number of admissions, aetiology, and pre- and post-social restrictions due to COVID-19, as well as demographics, location, and the severity of the social restrictions implemented. The extraction form was piloted using a sample of six randomly chosen studies and revised after discussion amongst authors. One author (MA) extracted the information of interest from the included studies using the final version of the data extraction form, and a second author (MH) double-checked 20% of the included items. In the case of missing or unclear data, we contacted the corresponding author of the study to provide additional information. Two studies were excluded from the meta-analysis during this process (both because relevant data was not available). For studies that reported multiple comparison periods, admissions in previous years were considered for the control period to control for potential seasonality effects.

### Data analysis

A meta-analysis was conducted with ReviewManager 5.3 and Stata16. We conducted meta-analyses of proportions, based on the pooled differences between the intervention and control conditions for each hypothesis. We conducted 19 separate meta-analyses with forest plots, for overall mortality, severity, mechanisms and location on injuries. The odds ratios (ORs) for mortality, mechanism of trauma, and location of trauma were calculated using the Mantel–Haenszel method with random effects model regardless of heterogeneity. We used I^2^ statistics and Cochran’s Q test to evaluate inter-study heterogeneity, which was deemed to be significant if *I*^2^ > 50% or *p* < 0.10. Descriptive statistics were used for demographic variables. To investigate the impact of differences in COVID-19 prevalence at the time of the studies, a subgroup analysis was conducted at the continent level. Results of this analysis are reported in the Appendix.

### Outcomes

Primary outcomes were number of admissions, mortality and clinical characteristics of patients admitted (i.e., average length of stay (LOS), patients requiring intensive care (ICU), patients requiring mechanical ventilation, ISS, Glasgow Coma Scale (GCS)), and trauma aetiology versus baseline. Secondary outcomes were patients’ demographics characteristics.

### Bias assessment

The quality of the observational studies was assessed using the Newcastle–Ottawa Score (NOS) [15]. The GRADE approach was used to assess the quality of evidence of estimates as high, moderate, low, or very low based on considerations of study design (observational studies are rated low confidence) [16]. See also Table A7 in the Appendix for a summary of all the checks conducted.

## Results

**Fig. 1:**
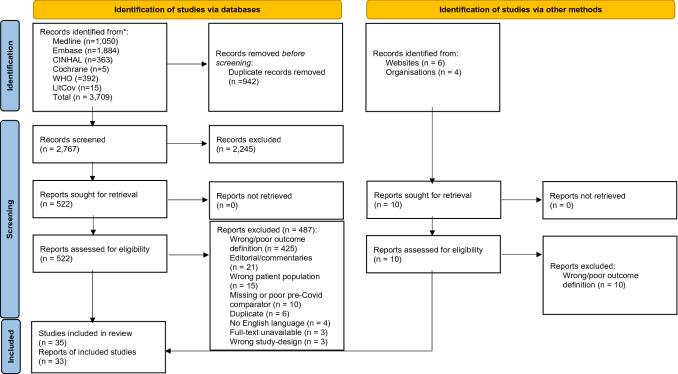
PRISMA flow diagram of studies selection process

The database search returned 3719 records in total. After removal of duplicates and limiting to language and publication type, 2777 records underwent title and abstract screening and 522 underwent full-text screening. 35 cohort studies were included in the analysis (see Fig. 1 [17]).

Table 1 reports the characteristics of the studies included in the analysis and the associated level of policy restrictions. The 35 studies included patients from 14 countries. The most represented countries are the US (*n* = 9), the UK (*n* = 4) and South Africa (*n* = 4). There were significant between-country differences in terms of the severity of the pandemic and the consequent stringency of the restrictions at the time of the study, both of which might affect the outcomes considered [18]. Therefore, the policy restrictions imposed at the time of the study were retrieved from the included articles, and collated as reported in the studies.

**Table 1:** Characteristic of the included studies

First author	Study design	Location	Sample size (pandemic vs control)	Pandemic period	Control period	Time (weeks)	Stringency level
Harris (2021)	Retrospective, single centre	Australia, Adelaide	193 vs 233	23 Mar–10 May 2020	3 Feb–22 Mar 2020–19	7	The South Australian government implemented 7 weeks of maximum social restrictions between 23 March–10 May 2020
Way (2020)	Retrospective, single centre (Level I Trauma centre)	Australia, Newcastle	259 vs 368	1 Mar–31 May 2020	1 Mar–31 May 2011–19	12	The New South Wales government introduced a nationwide lockdown from 29 March to 30 April 2020
Jacob (2020)	Retrospective, single centre study (level I trauma centre)	Australia, Sydney	37 vs 43.5	1 Mar–30 Apr 2020	1 Mar–30 Apr 2016–19	8	
Polan (2020)	Retrospective, single centre	Germany, Essen	22 vs 30	week 11–17 2020	week 11–17 2019	6	The German Government declared a temporary shutdown on Mar 16th 2020. On 22 March, the government and the federal states agreed for at least two weeks to forbid gatherings of more than two people. The national lockdown lasts until 6 May
Kreis (2021)	Retrospective, single centre (level 1 trauma centre)	Germany, Bonn	394 vs 524	16 Mar–19 Apr 2020	16 Mar–19 Apr 2019	5	
Ojetti (2020)	Retrospective, single centre (Level II Urban Teaching Hospital)	Italy, Rome	332 vs 813	21 Feb–31 Mar 2020	21 Feb–31 Mar 2019–18	6	With the Decree implemented on 9th March 2020, the government extended the lockdown and social distancing interventions homogeneously throughout the country. On 4th May 2020, the national exit strategy began
Christey (2020)	Retrospective, single centre (level I trauma centre)	New Zealand, Hamilton	71 vs 124	26 Mar–8 Apr 2020	5 Mar–18 Mar 2020	2	Declaration of alert level 2 on 19 March and declaration of the level 4 lockdown on 26 March 2020 by the New Zealand Government. New Zealand moves to Alert Level 1 on 8 June given the absence of cases in the country
Christey (2021)	Retrospective, multicentre	New Zealand, Midland region	1015 vs 1242	26 Mar–8 Jun 2020	26 Mar–8 Jun 2017–19	10	
McGuinness (2021)	Retrospective, multicentre	New Zealand, Northern Regions	123 vs 163	16 Mar–8 Jun 2020	16 Mar–8 June 2019	11	
Navsaria (2021)	Retrospective, single centre (tertiary urban trauma centre)	South Africa, Cape Town	628 vs 1328	Apr–May 2020	Feb–Mar 2020; Jun 2020	8	The national lockdown was announced on 23 March 2020 and came into effect at midnight on 26 March. On top of movement restrictions, the government implemented an absolute ban on sales of alcohol and tobacco products Easing of the lockdown regulations came into effect on 1 June 2020, including the resumption of the sale of alcohol
Venter (2020)	Retrospective, single centre (Emergency department—academic tertiary hospital)	South Africa, Guateng Province	3239 vs 4300	Feb–Jun 2020	Feb–Jun 2019	20	
Morris (2020)	Retrospective, single centre	South Africa, Kwa-Zulu Natal	706 vs 1337.5	Apr 2020	Apr 2019–18	4	
Zsilavecz (2020)	Retrospective, single centre	South Africa, Pietermaritzburg	154 vs 304	23 Mar–31 May 2020	23 Mar–31 May 2015–19	9	
Greenhalgh (2020)	Retrospective, single centre (a tertiary urban trauma centre)	UK, Lancashire	95 vs 147	16 Mar–22 Apr 2020	16 Mar–22 Apr 2019	6	Measures taken by the UK government included the implementation of social distancing on 16 March 2020 and escalated to advising the general population to stay at home if possible on 23 March 2020 implementing a national lockdown. On 10 May 2020, the PM announced a conditional plan for lifting lockdown announcing that people who cannot work from home should return to the workplace but avoid public transport
Rajput (2020)	Retrospective, single centre (level I major trauma centre)	UK, Liverpool	121 vs 194	23 Mar–10 May 2020	23 Mar–10 May 2019	7	
Ajayi (2020)	Retrospective, single centre (level I major trauma centre)	UK, London	217 vs 127	5 Mar–14 Apr 2020	1 Jan–5 Mar 2020	6	
Jeffries (2021)	Retrospective, single centre (major trauma centre)	UK, Belfast	42 vs 57	23 Mar–29 May 2020	23 Mar–29 May 2019	10	
Berg (2021)	Multicentre study from a large, multistate hospital network	US	4997 vs 7398	1 Apr–30 Apr 2020	1 Apr–30 Apr 2019	4	The individual states’ responses to COVID-19 varied initially from minimal response to stay-at-home (SAH) orders
Qasim (2020)	Retrospective, multicentre	US, Philadelphia	1058 vs 1328	9 Mar–19 Apr 2020	9 Mar–19 Apr 2019	6	From March 13, all Pennsylvania schools were closed. On March 16, the State governor implemented state-wide stay at home orders and the closure of all essential services. On April 1, a state-wide stay-at-home order across was implemented
Abdallah (2020)	Retrospective, single centre (level 1 trauma centre)	US, Philadelphia	480 vs 544	16 Mar–30 May 2020	16 Mar–30 May 2015–19	10	
Kamine (2020)	Retrospective, single centre (level II trauma centre)	US, Portsmouth (NH)	26 vs 33	16 Mar–4 Apr 2020	16 Mar–14 Apr 2017–19	4	Voluntary orders were imposed in New Hampshire to close schools on March 16, 2020, and to stay at home on March 27, 2020
Yeates (2020)	Retrospective, multi-centre study (level II trauma centres)	US, Southern California	6719 vs 7707	19 Mar–30 Jun 2020	19 Mar–30 Jun 2019	10	On March 19, 2020, the California Governor implemented a stay-at-home order, directing all Californians to remain at home except in cases of essential work or shopping for essential needs
Chiba (2021)	Retrospective, single centre (academic trauma centre)	US, Los Angeles	1202 vs 1143	20 Mar–30 Jun 2020	20 Mar–30 Jun 19	13	
Ghafil (2021)	Retrospective, multicentre (level 1 and 2 trauma centres)	US, Los Angeles County	6777 vs 6937	1 Jan–7 Jun 2020	1 Jan–7 Jun 2019	21	
Matthay (2021)	Retrospective, single centre (level 1 trauma centre)	US, San Francisco	749 vs 1580	18 Mar–17 Jul 2020	1 Jan–17 Mar 2020	16	
Devarakonda (2021)	Retrospective, single centre (academic level 1 trauma centre)	US, Augusta	574 vs 607	1 Mar–15 Jun 2020	1 Mar–15 Jun 2015–19	14	In Georgia, all public schools, colleges, and universities were close from March 18 through the start of April. On March 23, gatherings of over 10 people were banned, bars and nightclubs were ordered to close, and a shelter-in-place order for the "medically fragile" was issued. On April 2, a state-wide shelter in place order was issued
Walline (2021)	Retrospective, single centre (tertiary-care teaching hospital)	Hong Kong	382 vs 454	1 Jan–30 Jun 2020	2 Jan–30 Jun 2018–19	24	On 25 January, the Hong Kong government classified the COVID-19 with the maximum level of alert and shut down public venues and parks. All government employees were forced to work from home. From 30 January, borders closure with China started to be issued. On 27 Marc, the public health authorities banned indoor and outdoor public gatherings of more than four people
Moyer (2021)	Retrospective, multicentric cohort-based	France	361 vs 628	17 Mar–10 May 2020	17 Mar–10 May 2017–19	8	From March 13 2020, the French government imposed a first national lockdown for a duration of 55 days to reduce the spread of the virus
van Aert (2021)	Retrospective, single centre (level 2 trauma centre)	The Netherlands, Breda	36 vs 30	11 Mar–10 May 2020	11 Mar–10 May 2018–19	8	On 11 March 2020, the Dutch government introduced the first nationwide restrictive measures such as advice to limit the number of social contacts and to work from home). On 10 May 2020, these measures started to be lifted, by releasing the lockdown and opening primary schools
Nia (2021)	Retrospective, single centre (level 1 trauma centre)	Austria, Wien	43 vs 50	15 Mar–30 Apr 2020	15 Mar–30 Apr 2019	6	The Austrian government issued a nationwide lockdown from 14 March with full restrictions enforced two days after from 16 March
Riuttanen (2021)	Retrospective, multicentric cohort-based	Finland	47 vs 58	16 Mar–31 May 2020	16 Mar–31 May 2016–18	11	On March 16, 2020, the Finnish Government declared a state of emergency in response to the COVID-19 outbreak. All permanent residents were asked to minimize social contacts and to avoid non-essential travel and spending time in public places. Schools and educational institutions were closed down and face-to-face teaching was suspended
Kuo (2021)	Retrospective, single centre (level 1 trauma centre)	Taiwan	1955 vs 1650	1 Jan–31 Jun 2020	1 Jan–31 Jun 2015–19	26	Taiwan did not face a large amount of COVID-19 cases and the government did not adopt a lockdown strategy in the period considered. The only restrictive manners that the government applied to people were wearing a mask in public and social distancing
Rozenfeld (2021)	Retrospective, multicentric cohort-based	Israel	3997 vs 5439	15 Mar–30 Apr 2020	15 Mar–30 Apr 2016–19	6	In Israel, the Ministry of Health published on 15 March a list of restrictions to be enacted to arrest the spread of the virus. From 25 March, public gatherings and public transportation were terminated and only essential workers were allowed to leave their homes. From 4–5 May, the majority of normal activities were reopened
Hazra (2021)	Retrospective, single centre (level 1 trauma centre)	South India	30 vs 28*	1–30 Apr 2020	1–30 Apr 2018	4	On 24 March 2020, the Indian government issued a nationwide lockdown for 21 days which was extended till May 2020. All non-essential stores, activities and liquor shops were shut down
Quraishi (2021)	Retrospective, single centre	India, Nagpur	260 vs 784	25 Mar–31 May 2020	16 Jan–16 Mar 2020	9	

*Only ISS > 14 reported for this study due to lack of information on the overall trauma

### Major trauma admissions and clinical characteristics of patients admitted

**Fig. 2:**
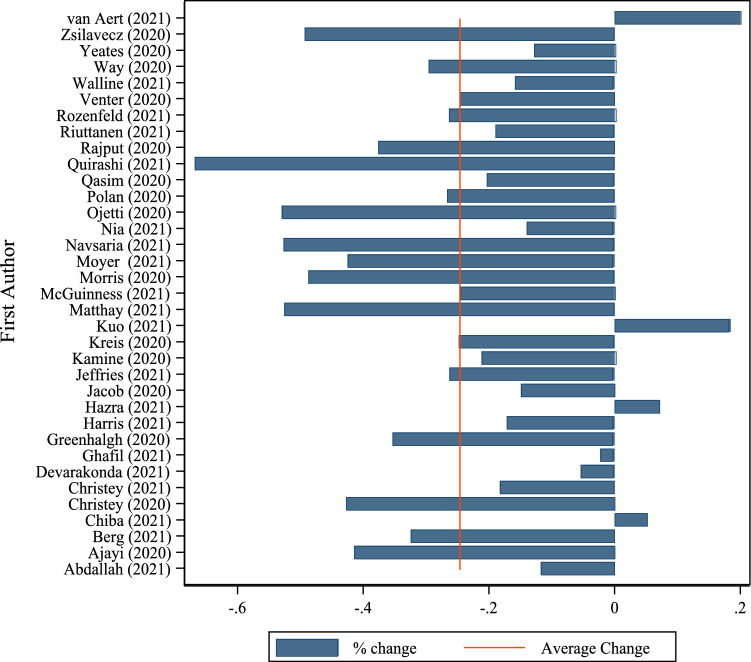
% variation of major trauma admissions pre-COVID-19 versus COVID-19

During the implementation of social restriction orders in response to the COVID-19 outbreak, there was a statistically significant reduction in major trauma presentations overall compared with control periods (mean −24%; *p* < 0.01; 95%CI [−0.31; −0.17]). The results are reported in Fig. 2. Only 4 studies out of 35 reported a positive percentage increase compared to the pre-restriction period [19–22] (see Figure A1 in the Appendix for a breakdown at the continent level).

Key clinical characteristics of the hospitalised cases are summarised in Table 2. Seventeen studies reported a measure of ISS for patients admitted to hospital [8, 10, 11, 22–35]. The median Injury Severity Score (ISS) remained unchanged in most of the studies between the two periods. We also collated the number of patients with an ISS > 12, which was reported in six studies [8, 11, 19, 23, 36, 37] (Figure A2 in the Appendix). The OR of the number of patients in this category before and during social movement restrictions derived from the meta-analysis was 1.17 (95% CI [0.77, 1.79]), suggesting no change in the number of severely injured patients. Morris et al.[38] reported similar results based on the South African Trauma Scale. There were two exceptions. Riuttanen and colleagues [29] reported a significant increase in the median ISS in four Finnish hospitals after the COVID-19 restrictions (18 vs 21, *p* = 0.008). In a study conducted in Israel, Rozenfeld et al. [39] found statistically significant variations by ISS groupings; a decrease for trauma admissions with an ISS 1–8, and an increase for ISS 9–14 and ISS 16–24, and no change for highly severe injuries (ISS 25–75).

**Table 2:** Clinical characteristics of major trauma

Authors	Median ISS post [IQR] (pre vs post)	Length of stays (mean or median) (SD) (pre vs post)	Patients requiring ICU (% of the total) (pre vs post)	Mechanical ventilation (% of the total) (pre vs post)	Ventilator days (mean or median) [IQR]	GCS (mean or median) (SD) (pre vs post)
Harris. (2021)	4 [NR] vs 4 [NR]	NR	NR	NR	NR	NR
Jacob (2020)	9 [4–17] vs 9 [4–17]	6.7 (± 0.4) vs 5.2 (± 6.5)	33 vs 39	NR	NR	NR
Christey (2020)	4 [6] vs 4 [8]	5.7 vs 3.8	5.6 vs 4	5.6 vs 2.8	2 [6] vs 1.5 [0.5] (median)	14 [NR] vs 14 [NR] (median)
Yeates (2020)	5 [1–10] vs 5 [1–10]	4.4 (± 8.58) vs 3.9 (± 5.67)	24 vs 22	NR	0.47 (± 2.56) vs 0.40 (± 2.27) (mean)	14.08 ± (2.53) vs 14.13 ± (2.43)
Way (2020)	8 [4–16] vs 9 [4–16]	3 vs 3 (median)	15 vs 13	3 vs 3.5	3 [2–5] vs 3 [2–6] (median)	15 [NR] VS 15 [NR] (median)
Abdallah (2020)	8.32 (± 10.22) 7.9 (± 8.84) (mean)	NR	NR	NR	NR	NR
Chiba (2021)	5 [2–11] vs 5 [1–10]	2 [NR] vs 2 [NR] (median)	31 vs 26	14.1 vs 9.2	3 [2–6] vs 3 [2–6] (median)	NR
Walline (2021)	9 [17] vs 10 [15.5]	10.3 (± 20.6) vs 10.4 (± 39)	17 vs 21	NR	NR	15 [NR] vs 15 [NR] (median)
Moyer (2021)	15.8 vs 16.4 (mean)	5.8 (± 14.8) vs 6.8 (± 13.8)	NR	NR	7.5 (± 11.6) vs 9.5 (± 15.9) (mean)	NR
Nia (2021)	12 (± 7.29) vs 16 (± 17.09) (mean)	NR	26 vs 23	NR	NR	NR
Riuttanen (2021)	18 [9] 21 [10]	2 [NR] vs 2 [NR] (median)	NR	NR	NR	NR
Rozenfeld (2021)	NR	NR	6 vs 6.3	NR	NR	NR
McGuinness (2021)	7.4 [NR] vs 6.8 [NR]	12 (± 16.7) vs 9 (± 8.5)	29 vs 30	NR	NR	NR
Berg (2021)	8.3 (7.5) vs 9.0 (7.5)	5.1 (± 8.2) vs 4.5 (± 5.1)	NR	NR	NR	14.3 (± 2.3) vs 14.2 (± 2.5) (mean)
Devarakonda (2021)	9 [NR] vs 9 [NR]	3.9 (± 6) vs 5.4 (± 9.6)	NR	NR	NR	NR
Quraishi (2021)	NR	3(1–12) vs 3(1–5) (median)	12.6 vs 12.3	8.2 vs 6.5	NR	NR
Ghafil (2021)	5 (2–13) vs 5 (2–13)	5.5 (4.82–6.26) vs 5.3 (4.67–5.99)	28.8 vs 28.5	28.9 vs 31.8	1.55 (1.21–1.98) vs 1.44 (1.13–1.83) (mean)	15 [14, 15] vs 15 [14, 15] (median)
Jefferies (2021)	27.8 vs 22 (mean)	NR	NR	NR	NR	NR
Matthay (2021)	5 [NR] vs 5 [NR]	1.4 (NR) vs 1.3 (NR)	26 vs 27	9.1 vs 10.9	0 [NR] vs 0 [NR] (median)	15 [NR] vs 15 [NR]

ISS: Injury Severity Score; IQR: Interquartile Range; NR: data not reported

The study included only severely injured patients with a ISS > 15

*Only ICU LOS reported

In terms of the LOS during movement restrictions, overall there was no significant difference in values compared to the pre-pandemic period (*n* = 10 studies; mean 0.52, *p* = 0.12). Two studies [27, 32] reported an increase in the LOS after the introduction of the restrictions. There was no statistically significant difference between the two periods in terms of patients admitted to the ICU (*n* = 12 studies; mean: −0.006, *p* = 0.25) (Figure A3 in the Appendix). Six studies reported information concerning patients requiring mechanical ventilation [8, 22, 23, 33, 35, 37]. A statistically significant reduction was reported for three studies [22, 23, 37], while the remaining three reported a statistically significant increase compared to the previous period [8, 33, 35]. No statistically significant difference was found for the number of days on a ventilator [8, 22–24, 27, 33, 35] or for the overall GCS score in any study [8, 23, 24, 26, 31, 33, 35].

### Mortality

**Fig. 3:**
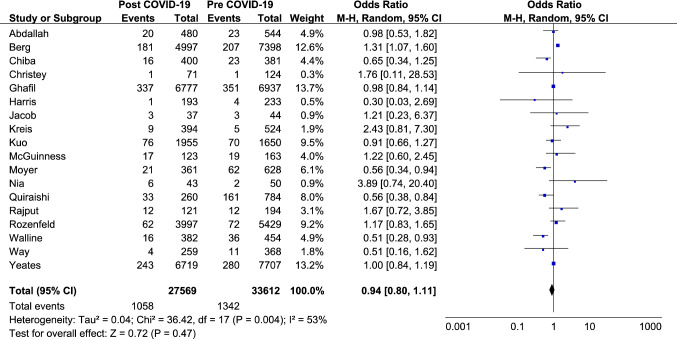
Mortality of major trauma admitted patients

Eighteen of the included studies reported the mortality of admitted patients summarised in Fig. 3. The studies provide no evidence for a change in mortality during the COVID-19 period (OR:0.94, 95%CI [0.80,1.11]). Three papers [24, 31, 33], all run in the US, had a weight greater than 10% in the analysis. When we ran the analysis excluding these three publications, the results were consistent with the primary analysis (OR:0.87, 95%CI [0.68,1.11]). The subgroup analysis by continent confirms this, excepting countries in Asia, where three studies [20, 26, 37] reported a statistically significant reduction in the period considered (OR:0.67, 95%CI [0.46, 0.98]) (Figure A4 in the Appendix).

### Mechanisms of injury

Table 3 reports the distribution of injury mechanisms in the included studies. There was an overall statistically significant reduction in trauma due to motor vehicle/road traffic collisions (OR:0.70;95%CI [0.61,0.81])[8, 10, 11, 22–43]. A sensitivity analysis showed that this outcome was not statistically significant in South Africa and Asian countries (see Figure A5 in the Appendix). There was no statistically significant difference between the two periods for motorbike (OR:0.89; 95%CI [0.73,1.08]) [10, 11, 22–24, 29, 33–36, 39, 40, 42] or bicycle collisions (OR:1.08; 95%CI [0.80, 1.47]) [10, 11, 22, 23, 29, 30, 34–36, 39, 40, 42]; however, there was a statistically significant reduction observed for pedestrian collisions (OR:0.63; 95%CI [0.51,0.78]) [10, 11, 22–25, 29, 30, 33–36, 38–40]. An overall statistically significant reduction (OR:0.66; 95%CI [0.46, 0.94]) was observed for major trauma admissions due to assault during the period of restrictions in sixteen studies [8, 10, 11, 20, 22, 24, 25, 27–30, 33, 36, 37, 40, 44]. Among these, three studies [8, 20, 37] reported a statistically significant reduction, while the others reported a non-statistically significant reduction or a stable trend over time [10, 11, 22, 24, 25, 27–30, 33, 35, 36, 40]. The subgroup analysis showed that a statistically significant reduction was observed only in countries in Asia (Figure A6 in the Appendix). There was an overall statistically significant increase in admissions due to firearms and gunshot wounds (OR:1.34; 95%CI [1.11,1.61]) across thirteen studies [10, 22, 24, 25, 29, 31, 33, 35, 38, 40, 41, 43, 44]. Among these, only one study [38] carried out in a single centre hospital in South Africa reported a statistically significant reduction in firearm and gunshot wounds (Figure A7 in the Appendix). There was an increasing trend for stab wound admissions in nine studies, but the value was not statistically significant (OR:1.12; CI [0.98,1.28]) [22, 24, 25, 32, 33, 41–44]. There was no statistically significant difference for falls (see also Figure A8 in the Appendix) and other mechanisms which comprises minor injury mechanisms such as dog bites, found down, and finger trapped. An overall statistically significant increase in admissions due to suicide attempts or self-harm was observed across thirteen studies (OR:1.41; 95%CI [1.05, 1.89]) [8, 10, 11, 20, 22, 24, 28–30, 35, 36, 40, 42] (Figure A9 in the Appendix). Among these, only four studies [7, 11, 30, 36] reported a decreasing trend, which was not statistically significant in any study.

**Table 3:** Injury mechanisms

	Odds ratio	Heterogeneity I^2^	Mean (%) (pre vs post)	Studies	Quality of evidence
Mechanism of trauma					
Motor vehicle/road traffic-related	0.70 [0.61, 0.81], *p* = < 0.01*	91% (*p* = < 0.001)	30 vs 24	25	Moderate†
Motorbike	0.89 [0.73, 1.08], *p* = 0.24	72% (*p* = < 0.001)	10 vs 8	13	Low
Pedestrian	0.63 [0.51, 0.78], *p* = < 0.01*	79% (*p* = < 0.001)	7 vs 5	15	Moderate†
Bicycle	1.08 [0.80, 1.47], *p* = 0.62	71% (*p* = < 0.001)	7 vs 6	12	Low
Assault	0.66 [0.46, 0.94], *p* = 0.02*	93% (*p* = < 0.001)	13 vs 8	16	Low^‡^
Firearm and gunshot	1.34 [1.11, 1.61], *p* = < 0.01*	73% (*p* = 0.02)	5 vs 7	13	Moderate†
Stab wound	1.12 [0.98, 1.28], *p* = 0.1	59% (*p* = 0.01)	11 vs 11	9	Low
Self-inflicted/suicide attempts	1.41 [1.05, 1.89], *p* = 0.02*	39% (*p* = 0.07)	3 vs 6	13	Moderate†
Falls	1.08 [0.96, 1.21], *p* = 0.20	83% (*p* = < 0.001)	32 vs 35	18	Low
-Falls from standing	1.01 [0.95, 1.07], *p* = 0.77	35% (*p* = 0.14)	19 vs 20	9	Low
-Falls from height	1.18 [0.90, 1.55], *p* = 0.22	76% (*p* = < 0.001)	10 vs 13	9	Low
Other (found down, dog bites, trapped finger, law enforcement, etc.)	1.24 [0.98, 1.58], *p* = 0.08	71% (*p* = < 0.001)	7 vs 9	15	Low
Location of trauma					
Road	0.70 [0.56, 0.89], *p* = < 0.01*	87% (*p* = < 0.001)	38 vs 31	12	Moderate†
Home	1.51 [1.17, 1.94], *p* = < 0.01*	91% (*p* = < 0.001)	30 vs 41	11	Moderate†
Other (outdoor, workplace, etc.)	0.73 [0.59, 0.89], *p* = < 0.01*	64% (*p* = 0.004)	21 vs 17	9	Low

*Statistically significant at the 95% confidence level

The quality of evidence was assessed using the GRADE approach

High quality: We are very confident that the true effect lies close to that of the estimate of the effect

Moderate quality: We are moderately confident in the effect estimate: The true effect is likely to be close to the estimate of the effect, but there is a possibility that it is markedly different

Low quality: Our confidence in the effect estimate is limited: The true effect may be markedly different from the estimate of the effect

Very low quality: We have very little confidence in the effect estimate: The true effect is likely to be markedly different from the estimate of effect

^†^Updated due to large magnitude of effect

^‡^Update due to large magnitude effect; reduced due to limitations in study design or execution (risk of bias)

In terms of location, there was a statistically significant reduction in road trauma across twelve studies (OR:0.70; 95%CI [0.56, 0.89]) [10, 11, 20, 23, 28, 30, 36, 39, 41, 42] and in other locations that imply individuals to be outside the home, such as outdoor or workplace, grouped under the label “Other” due to the high heterogeneity in the way in which they were reported (OR:0.73; 95%CI [0.59,0.89]) [10, 20, 23, 26, 30, 36, 40–42] (Figure A10 in the Appendix). There was a statistically significant increase in admissions for trauma occurring at home across eleven studies (OR:1.51; 95%CI [1.17, 1.94]) [10, 20, 23, 24, 26, 28, 30, 36, 39, 40, 42] (Figure A11 in the Appendix). Among these, only one study [30] reported a statistically significant reduction during the societal restrictions period. The increase is not only the result of a larger relative proportion of total presentations with respect to the reduction in presentations due to road collisions, but also an increase in the absolute numbers of trauma occurring at home compared to the previous period across studies [20, 28, 36, 39, 40]. By comparing the average home trauma admissions across studies before and after the pandemic, we found an overall average increase in the pandemic period of approximately 7%.

### Demographics

We found no statistically significant differences for the average age and the share of females on the total number of admitted patients (*p* > 0.2) (see Table 4). Five studies [11, 23, 36, 37, 44] reported a significant increase in the proportion of females on the total, while three studies [25, 29, 34] reported a statistically significant reduction. Only one study [29] reported a clear and significant decline in the average age of patients admitted during the COVID-19 period compared to the pre-pandemic period.

**Table 4:** Demographic information of patients admitted due to major trauma

First author	Female (%) pre vs post	Age—(mean or median) (SD) pre-covid	Age (mean or median) (SD) post-covid
Harris (2021)	27 vs 25	NR	NR
Way (2020)	27 vs 29	40 (± 23)	38 (± 21)
Jacob (2020)	21 vs 27	39.81 (± 18.3)	43.87 (± 21.3)
Polan (2020)	NR	NR	NR
Ojetti (2020)	NR	NR	NR
Christey (2020)	32 vs 41	37.4 (± 25.6)	40.9 (± 25.9)
Navsaria (2021)	NR	NR	NR
Venter (2020)	NR	NR	NR
Morris (2020)	NR	NR	NR
Zsilavecz 2020	19 vs 24	NR	NR
Greenhalgh (2020)	NR	NR	NR
Rajput (2020)	25 vs 21	41 (Median)	36 (Median)
Ajayi (2020)	40 vs 40	NR*	NR*
Qasim (2020)	NR	NR	NR
Kamine (2020)	NR	NR	NR
Yeates (2020)	33 vs 33	47.16 (± 24.1)	46.43 (± 23.9)
Abdallah (2020)	32 vs 28	46 (± 21)	42.90 (± 20.3)
Chiba (2021)	21 vs 23	38 (Median)	40 (Median)
Walline (2021)	34 vs 33	52.4 (± 24.5)	53 (± 23.9)
Moyer (2021)	22 vs 18	41.5 (± 19)	43.2 (± 20)
van Aert (2021)	NR	NR	NR
Nia (2021)	26 vs 30	42.88 (± 27.3)	45.19 (± 26.9)
Riuttanen (2021)	58 vs 47	53 (± 19)	47 (± 21)
Kuo (2021)	37 vs 38	49 (± 23.4)	50 (± 22.9)
Rozenfeld (2021)	40 vs 39	NR*	NR*
Matthay (2021)	NR*	NR*	NR*
McGuinness (2021)	24 vs 27	44.1 (± 22.3)	48.8 (± 23.3)
Christey (2021)	27 vs 29	40 (± 23)	38 (± 21)
Berg (2021)	46 vs 43	NR*	NR*
Quraishi (2021)	12 vs 16	NR	NR
Kreis (2021)	41 vs 42	NR	NR
Ghafil (2021)	26 vs 25	45 (± 20)	45 (± 20)
Jefferies (2021)	33 vs 24	38 (± 21)	32 (± 18)
Hazra (2021)	NR	NR*	NR*

NR: data not reported

*Reported in a different format

Not reported for major trauma only

### Risk of bias assessment

Table A8 (supplementary appendix) reports the risk of bias assessment. Overall, 26 out 35 (74%) of the included studies were considered to be at low risk of bias [8, 10, 11, 20, 22, 24–33, 35–39, 41–46]. Two studies [12, 47] were classified as being at a high risk of bias, primarily due to a lack of comparability with other studies and definition of the outcomes of interest, and were therefore excluded from the meta-analysis. These studies were included in the descriptive figures and in the demographic table. The remaining seven studies reported a low/moderate risk of bias [19, 21, 23, 34, 40, 48, 49]. With further informal screening to assess the comparability of outcomes and potential biases in the population included, these were included in the meta-analysis. Less than half of the studies used at least two years of prior data to ascertain a control period [8, 10, 11, 20, 25–27, 29, 32, 36, 38, 39, 44, 47, 48]. The average length for the control period in the included studies was 2.2 years (± 1.8), with 45% of the studies included using a historical control based on data from more than 1 year, and 31% more than 2 years.

## Discussion

From our analysis, two of our three a priori hypotheses were supported: overall, we observed a significant reduction in major trauma presentations and a reduction in traffic-related injuries alongside an increase in trauma occurring at home during the COVID-19 restrictions compared to the pre-pandemic period. However, we did not observe an increase in severely injured patients with the introduction of social restrictions. Further, we did not observe a significant change in mortality of the admitted patients. Findings from the subgroup analysis signal that most of the significant variations were recorded in the continents broadly most affected by the virus, which may signal an incremental marginal effect of the virus on major trauma epidemiology.

As expected, policy restrictions resulted in reduced road collisions and those in other locations such as outdoor places and workplaces [20, 36, 44] and an overall statistically significant increase in trauma occurring in the homes.

Social and movement restrictions have previously been shown to disproportionally affect low-income families and workers in terms of reduced income and increased social isolation [50, 51]. Others have suggested that the loss of financial stability and forced social isolation policies might lead to an increase in intentional injury and violence [24, 25, 52]. Our results show an overall statistically significant reduction in assault trauma and mixed results in terms of stab wounds [22, 24, 25, 32, 33, 35, 41–44]. However, we did find a statistically significant increase in trauma presentations resulting from firearms and gunshot wounds, in line with previous findings from the literature [25, 52]. This divergence with assault and stab trauma can be explained with additional analysis. First of all, it is a relative increase, meaning that the total number of events has remained stable compared to the pre-pandemic period while other trauma typologies dropped in all countries. Secondly, the result is mainly driven by data from the US (46% of all the studies), where firearm legislation is relatively liberal, and from South Africa (30% of the total), where interpersonal violence and firearms traumas are a significant issue [35, 38, 40]. In the other countries, where firearm injuries pre-COVID-19 were rare, we did not find any significant variation between the two periods. We were unable to systematically examine traumas due to domestic violence as only three studies [24, 28, 40] reported this measure, all of which reported a similar proportion compared to the pre-pandemic period.

A number of authors have previously raised concerns relating to the potential impact of prolonged lockdowns and social restrictions on mental health outcomes, such as suicide rates and self-harm [53, 54]. Previous findings reported mixed evidence compared to the pre-pandemic period [55–57]. We observed a statistically significant increase in trauma presentations due to suicide attempts and self-harm during social movement restrictions. We observed both a relative (53% of the total cases in the COVID-19 period, compared to 33% in the pre-pandemic period) and an absolute increase (average increase of 2 per study) across thirteen studies [8, 10, 20, 22, 24, 28–30, 35, 36, 40, 42]. The four studies that reported a decreasing (not statistically significant) trend were conducted in New Zealand and Australia, where the impact of COVID-19 has been minimal, as well as the corresponding stringency of containment measures for much of 2020 [4]. Additional research is required to infer whether the effects of the pandemic itself, or the containment measures was more relevant for the trend observed.

Several limitations need to be acknowledged in our analysis. Firstly, most of the included studies provided data on a relatively short time interval both pre- and post-comparison, which may not be adequate observation time to ascertain the true effects of a pandemic on trauma presentations. Extensive evidence reports that trauma epidemiology changes over a short period of time must be interpreted in the setting of normal seasonal variations [58, 59]. Secondly, this meta-analysis also demonstrated a high degree of statistical heterogeneity in the primary outcomes (I^2^ statistic ranging from 39 to 91%) which most likely originates from the clinical heterogeneity within trauma cohorts, the different time periods considered, the local intensity of SARS-CoV-2 infection, and the extent of social movement restrictions within the included studies. We attempted to explore this heterogeneity through a subgroup analysis at the continent level.

Additionally, most of the included studies did not report whether a hospital was a designated COVID-19 facility. Only two studies [28, 31] reported a change in the trauma destination protocols in response to public health priorities. Self-presentations from injury may decrease in a COVID-designated facility. This lack of information may bias our conclusions, as 72% of the studies included in the meta-analysis are single-centre studies and are therefore more susceptible to such changes. Finally, we only included articles published in English, and therefore, some relevant references published in other languages will have been excluded. Future research should fill this gap and confirm our findings, possibly comparing major trauma admissions across different waves of COVID-19.

This review is the first article that systematically analyses the impact of policy restrictions for pandemic control, and the pandemic itself, on major trauma epidemiology. The results of this systematic review provide relevant information for policy-makers about the implications of disassembling or reducing trauma services, redeploying staff to other tasks, and hospital financing needs, to inform responses to future public health crises when a novel virus is emerging, and vaccines are not yet available. Although new information continues to develop, this review reports evidence of an absolute volume reduction in major trauma admissions with unchanged severity and mortality during the first wave of COVID-19 movement restriction policies. Current data based on the first wave of the COVID-19 do not support the reallocation of highly specialised trauma professionals. If trauma professionals were redeployed to pandemic management at a higher rate than the corresponding reduction in major trauma admissions, there may be a greater burden for trauma professionals with a potential increase in the risk of morbidity and mortality for patients. The described epidemiological changes are essential to inform resource allocation decisions in future waves of viral pandemics and to identify the trauma mechanisms for which hospital and community investments and prevention programs are most needed during public health emergencies.

## Supplementary Information

Below is the link to the electronic supplementary material.

Supplementary file1 (DOCX 2221 kb)
