# Supplementary material for: The Epidemiology of Major Trauma During the First Wave of COVID-19 Movement Restriction Policies: A Systematic Review and Meta-analysis of Observational Studies
Source: World J Surg. 2022 Jun 20;46(9):2045–60. doi: 10.1007/s00268-022-06625-7 (PMC9208248; doi:10.1007/s00268-022-06625-7)
Supplement: Supplementary file 1 — Supplementary file1 (DOCX 2221 kb) [file 268_2022_6625_MOESM1_ESM.docx]

**Appendix – Supplementary material**

Table A1 - Search strategy example – Medline and Lit COVID search

| 1. exp Coronavirus/ or coronavirus.mp. |
| --- |
| 2. Covid-19.rx. or Covid-19/ |
| 3. exp Coronavirus infections/ |
| 4. (OC43 or NL63 or 229E or HKU1 or HCoV* or ncov* or sars-cov* or sarscov* or Sars-coronavirus* or cov-2).mp. |
| 5. corona virus*.mp. |
| 6. covid*.mp. |
| 7. quarantine.mp. or Quarantine/ |
| 8. lockdown*.mp. |
| 9. stay*-at-home.mp. |
| 10. social distanc*.mp. |
| 11. (soci* restriction* or containment measure* or containment strategy* or shelter-in-place).mp. |
| 12. 1 or 2 or 3 or 4 or 5 or 6 or 7 or 8 or 9 or 10 or 11 |
| 13. major trauma*.mp. |
| 14. Trauma* injur*.mp. |
| 15. Fractures, Bone/ or Orthopedic procedures/ or "Wounds and Injuries"/ or Wound*.mp. or fracture*.mp. |
| 16. 13 or 14 or 15 |
| 17. volume*.mp. |
| 18. patient*.mp. |
| 19. demand*.mp. or "Health Services Needs and Demand"/ |
| 20. activit*.mp. |
| 21. impact.mp. |
| 22. trend*.mp. |
| 23. provision*.mp. |
| 24. (intervention* or presentation* or admission*).mp. |
| 25. (workload* or work load*).mp. or Workload/ |
| 26. Mortality/ or Cause of death/ or Survival rate/ or mortalit*.mp. |
| 27. Intensive Care Units/ or Trauma Centers/ or Critical Care/ |
| 28. (icu or intensive care or trauma center* or trauma centre* or critical care).mp. |
| 29. operation*.mp. |
| 30. complication*.mp. |
| 31. Treatment outcome/ or Trauma severity indices/ or Injury severity score/ |
| 32. Outcome*.mp. |
| 33. 17 or 18 or 19 or 20 or 21 or 22 or 23 or 24 or 25 or 26 or 27 or 28 or 29 or 30 or 31 or 32 |
| 34. 12 and 16 and 33 |
| 35. limit 34 to yr="2020 -Current" |

Table A2 - Search strategy example – Embase database

| 1. | coronavirus.mp. or exp Coronavirinae/ or Covid-19/ |
| --- | --- |
| 2. | coronavirus infection/ or severe acute respiratory syndrome/ |
| 3. | (OC43 or NL63 or 229E or HKU1 or HCoV* or ncov* or sars-cov* or sarscov* or Sars-coronavirus* or cov-2).mp. |
| 4. | (corona virus* or covid*).mp. |
| 5. | quarantine.mp. or communicable disease control/ or quarantine/ |
| 6. | lockdown*.mp. |
| 7. | Stay* at home.mp. |
| 8. | social distanc*.mp. |
| 9. | (soci* restriction* or containment measure* or shelter-in-place).mp. |
| 10. | 1 or 2 or 3 or 4 or 5 or 6 or 7 or 8 or 9 |
| 11. | major trauma*.mp. |
| 12. | Trauma* injur*.mp. |
| 13. | exp fracture/ or exp orthopedic surgery/ or wound/ or injury/ep, et, su or fracture*.mp. or wound*.mp. |
| 14. | 11 or 12 or 13 |
| 15. | volume*.mp. |
| 16. | Patient*.mp. |
| 17. | demand*.mp. |
| 18. | activit*.mp. |
| 19. | impact.mp. |
| 20. | trend*.mp. |
| 21. | provision*.mp. |
| 22. | (intervention* or presentation* or admission*).mp. |
| 23. | (workload* or work load*).mp. or Workload/ |
| 24. | Mortality/ or "cause of death"/ or survival rate/ or mortalit*.mp. |
| 25. | Intensive Care Unit/ or Emergency Health Service/ or Intensive Care/ |
| 26. | (icu or intensive care or trauma center* or trauma centre* or critical care).mp. |
| 27. | operation*.mp. |
| 28. | complication*.mp. |
| 29. | Treatment outcome/ or Injury scale/ |
| 30. | Outcome*.mp. |
| 31. | 15 or 16 or 17 or 18 or 19 or 20 or 21 or 22 or 23 or 24 or 25 or 26 or 27 or 28 or 29 or 30 |
| 32. | 10 and 14 and 31 |
| 33. | limit 32 to yr="2020 -Current" |

Table A3 - Search strategy example – CINHAL database

| 1 | (MH "Coronavirus Infections") or "covid*" or covid-19/ |
| --- | --- |
| 2 | OC43 or NL63 or 229E or HKU1 or HCoV* or ncov* or sars-cov* or sarscov* or Sars-coronavirus* or cov-2 |
| 3 | "corona virus" |
| 4 | "quarantine" or "isolation" or "social distanc*" or "lockdown*" or "soci* restriction*" or "containment measure*" or “containment strategy*” or "shelter-in-place" or (MH "Stay-at-Home Orders ") or (MH "Social Distancing") |
| 5 | (MH "Coronavirus") |
| 6 | S1 OR S2 OR S3 OR S4 OR S5 |
| 7 | "major trauma*" |
| 8 | "trauma* injur*" |
| 9 | "fracture*" OR (MH "Wounds and Injuries") OR (MH "Fractures") |
| 10 | S7 OR S8 OR S9 |
| 11 | "volume*" |
| 12 | "patient*" |
| 13 | "demand*" OR (MH "Health Services Needs and Demand/TD") |
| 14 | "activit*" |
| 15 | impact* |
| 16 | "trend*" |
| 17 | "provision*" |
| 18 | "intervention*" or "presentation*" or "admission*" |
| 19 | (MH "Workload") OR workload* or work load* or demand* or pressure |
| 20 | (MH "Intensive Care Units/TD/UT") OR (MH "Critical Care") OR (MH "Trauma Centers") OR "intensive care unit or icu or critical care or trauma center* or trauma centre*” |
| 21 | "mortalit*" OR (MH "Mortality")(MH "Cause of Death") |
| 22 | "operation*" |
| 23 | "complication*" OR "outcome*" |
| 24 | S11 OR S12 OR S13 OR S14 OR S15 OR S16 OR S17 OR S18 OR S19 OR S20 OR S21 OR S22 OR S23 |
| 25 | S6 AND S10 AND S24 |

Table A4 - Search strategy example – Cochrane Library

| 1 | MeSH descriptor: [Coronavirus] explode all trees |
| --- | --- |
| 2 | coronavirus or COVID-19 or corona virus or covid* |
| 3 | MeSH descriptor: [Coronavirus Infections] explode all trees |
| 4 | OC43 or NL63 or 229E or HKU1 or HCoV* or ncov* or sars-cov* or sarscov* or Sars-coronavirus* or cov-2 |
| 5 | quarantine or isolation or social distanc* or lockdown or Containment measure* or Shelter-in-place or stay* at home |
| 6 | MeSH descriptor: [Quarantine] |
| 7 | #1 OR #2 or #3 OR #4 OR #5 OR #6 |
| 8 | major trauma* |
| 9 | Trauma* injur* |
| 10 | fracture* or wound* |
| 11 | MeSH descriptor: [Orthopedic Procedures] explode all trees |
| 12 | MeSH descriptor: [Wounds and Injuries] explode all trees |
| 13 | #8 OR #9 OR #10 OR #11 OR #12 |
| 14 | Volume* |
| 15 | patient* |
| 16 | demand* |
| 17 | activit* |
| 18 | impact* |
| 19 | trend* |
| 20 | provision* |
| 21 | intervention* or presentation* or admission* |
| 22 | MeSH descriptor: [Workload] |
| 23 | workload or work load |
| 24 | MeSH descriptor: [Mortality] |
| 25 | MeSH descriptor: [Cause of Death] |
| 26 | MeSH descriptor: [Survival Rate] explode all trees |
| 27 | mortalit* |
| 28 | MeSH descriptor: [Trauma Centers] |
| 29 | MeSH descriptor: [Critical Care] |
| 30 | icu or intensive care or trauma center* or critical care |
| 31 | operation* |
| 32 | complication* |
| 33 | MeSH descriptor: [Treatment Outcome] |
| 34 | MeSH descriptor: [Trauma Severity Indices] |
| 35 | MeSH descriptor: [Injury Severity Score] |
| 36 | Outcome* |
| 37 | #14 OR #15 OR #16 #17 OR #18 OR #19 OR #20 OR #21 OR #22 OR #23 OR #24 OR #25 OR #26 OR #27 OR #28 OR #29 OR #30 OR #31 OR #32 OR #33 OR #34 OR #35 OR #36 OR #37 |
| 38 | #7 and #13 and #38 Between Jan 2020 and Aug 2021 |

Table A5 - Search strategy example – WHO literature

| mj:(covid OR quarantine OR "Quarantine" OR "Coronavirus Infections" OR "Pandemics" OR "Social Isolation" OR "Communicable Disease Control" OR "Coronavirus" OR "Social Distance") OR (coronavirus OR covid-19 OR sars-cov* OR corona virus OR covid* OR oc43 OR nl63 OR 229e OR hku1 OR hcov* OR ncov* OR sars-cov* OR sarscov* OR sars-coronavirus* OR cov-2 OR coronavirus infection* OR quarantine OR lockdown* OR stay at home OR social distanc* OR social soci* restriction* OR containment measure* OR containment strateg* OR shelter-in-place) AND mj:(trauma OR fracture OR orthopedic surgery OR "Wounds and Injuries") OR (traum* interventention* or fracture* or major traum*) AND mj:("Trauma Centers" OR mortality OR Trauma Severity Indices OR Injury Severity Score OR Cause of Death OR Survival Rate) OR (volume* OR patient* OR demand* OR activit* OR impact* OR trend* OR provision* OR intervention* OR presentation* OR admission* OR workload* OR work load* OR mortalit* OR intensive care unit OR icu OR trauma center* OR trauma centre* OR critical care OR operation* OR complication* or outcome*) |
| --- |

Table A6 - Summary of the inclusion criteria

| **Population** | **Intervention** | **Control** | **Outcomes** |
| --- | --- | --- | --- |
| Patients requiring trauma resuscitation based on institutional criteria on arrival to the emergency department. | Societal restrictions as response to the COVID-19 outbreak | Pre-pandemic, pre-restriction period | Number of admissions,  Clinical outcomes,  Aetiology,  Demographics, |

Table A7: MOOSE Checklist

| **Item No** | **Recommendation** | **Reported on Page No** |
| --- | --- | --- |
| Reporting of background should include | | |
| 1 | Problem definition | 2 |
| 2 | Hypothesis statement | 2 |
| 3 | Description of study outcome(s) | 3-4 |
| 4 | Type of exposure or intervention used | 3-4 |
| 5 | Type of study designs used | 3-4 |
| 6 | Study population | 4 |
| Reporting of search strategy should include | | |
| 7 | Qualifications of searchers (eg, librarians and investigators) | 5 |
| 8 | Search strategy, including time period included in the synthesis and key words | 5, Table A1,A2, A3, A4, A5 |
| 9 | Effort to include all available studies, including contact with authors | 4-5 |
| 10 | Databases and registries searched | 4-5 |
| 11 | Search software used, name and version, including special features used (eg, explosion) | 4-5 |
| 12 | Use of hand searching (eg, reference lists of obtained articles) | 4-5 |
| 13 | List of citations located and those excluded, including justification | Figure 1 |
| 14 | Method of addressing articles published in languages other than English | 5 |
| 15 | Method of handling abstracts and unpublished studies | 5 |
| 16 | Description of any contact with authors | 4, 6 |
| Reporting of methods should include | | |
| 17 | Description of relevance or appropriateness of studies assembled for assessing the hypothesis to be tested | 5-6 |
| 18 | Rationale for the selection and coding of data (eg, sound clinical principles or convenience) | 5 |
| 19 | Documentation of how data were classified and coded (eg, multiple raters, blinding and interrater reliability) | 5 |
| 20 | Assessment of confounding (eg, comparability of cases and controls in studies where appropriate) | 6 |
| 21 | Assessment of study quality, including blinding of quality assessors, stratification or regression on possible predictors of study results | 6 |
| 22 | Assessment of heterogeneity | 6 |
| 23 | Description of statistical methods (eg, complete description of fixed or random effects models, justification of whether the chosen models account for predictors of study results, dose-response models, or cumulative meta-analysis) in sufficient detail to be replicated | 5-6 |
| 24 | Provision of appropriate tables and graphics | - |
| Reporting of results should include | | |
| 25 | Graphic summarizing individual study estimates and overall estimate | Figs 2-3 |
| 26 | Table giving descriptive information for each study included | Table 1 |
| 27 | Results of sensitivity testing (eg, subgroup analysis) | - |
| 28 | Indication of statistical uncertainty of findings | 7-12 |

Source: [1] Stroup DF, Berlin JA, Morton SC, et al. Meta-analysis of observational studies in epidemiology: a proposal for reporting. Meta-analysis Of Observational Studies in Epidemiology (MOOSE) group. JAMA. 2000;283(15):2008-2012. doi:10.1001/jama.283.15.2008

**Subgroup analysis**

A sensitivity analysis grouping studies by continent is conducted to account for variations in the prevalence of the disease across countries during the period considered. Specifically, we grouped studies as: Asia (n=4), Europe (n=12, including Israel), and Oceania (n=5). In Africa, we only retrieved study from South Africa (n=4). Accordingly, we group them as a single country. Similarly, for North America we retrieved studies only from the US (n=9) and group them as a single country. No studies are retrieved from South America.

This subgroup analysis allows us to control both for the prevalence of the disease and for the regional variation. Indeed, Europe and the US were the area most severely affected throughout the first wave of the COVID-19, while South Africa and Asian countries were able to delay the impact of the first wave through countrywide lockdowns [2, 3].

Table A8: Risk of bias assessment

|  | **Selection** | | | | **Comparability** | | | **Outcomes** | | |
| --- | --- | --- | --- | --- | --- | --- | --- | --- | --- | --- |
| **Low risk** | 3 or 4 points | | | | 1 or 2 points | | | 2 or 3 points | | |
| **Moderate risk** | 2 points | | | | 1 or 2 points | | | 2 or 3 points | | |
| **High risk** | 0 or 1 point | | | | 0 point | | | 0 or 1 point | | |
|  | **Representativeness of the exposed cohort** | **Selection of the non-exposed cohort** | **Ascertainment of exposure** | **Demonstration that outcome of interest was not present at start of study** | **Comparability of cohorts on the basis of the design or analysis** | | **Based on mechanisms of the trauma** | **Assessment of outcome** | **Was follow-up long enough for outcomes to occur** | **Adequacy of follow up of cohorts** |
| Harris (2021) | 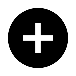 | 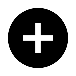 | 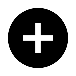 | 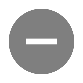 | 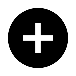 | 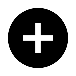 | | 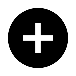 | 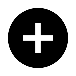 | 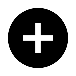 |
| Christey (2020) | 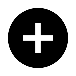 | 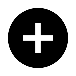 | 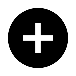 | 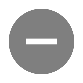 | 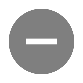 | 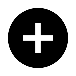 | | 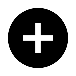 | 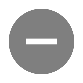 | 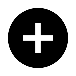 |
| Navsaria (2021) | 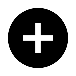 | 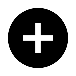 | 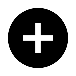 | 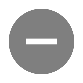 | 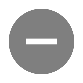 | 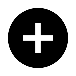 | | 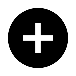 | 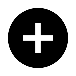 | 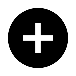 |
| Greenhalgh (2020) | 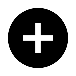 | 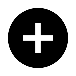 | 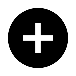 | 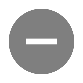 | 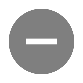 | 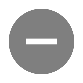 | | 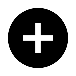 | 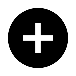 | 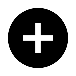 |
| Morris (2020) | 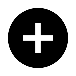 | 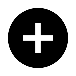 | 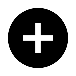 | 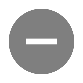 | 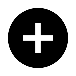 | 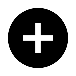 | | 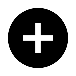 | 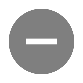 | 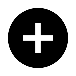 |
| Kamine (2020) | 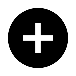 | 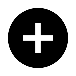 | 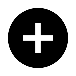 | 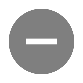 | 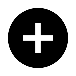 | 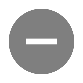 | | 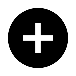 | 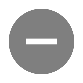 | 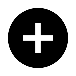 |
| Polan (2020) | 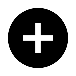 | 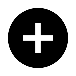 | 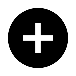 | 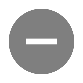 | 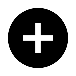 | 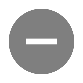 | | 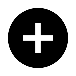 | 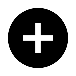 | 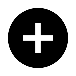 |
| Jacob (2020) | 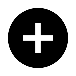 | 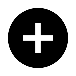 | 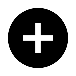 | 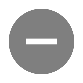 | 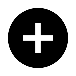 | 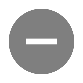 | | 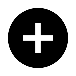 | 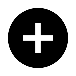 | 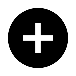 |
| Zsilavecz 2020 | 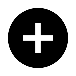 | 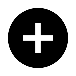 | 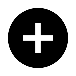 | 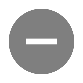 | 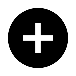 | 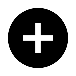 | | 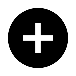 | 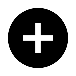 | 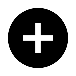 |
| Ajayi (2020) | 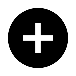 | 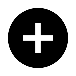 | 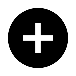 | 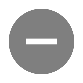 | 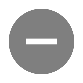 | 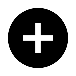 | | 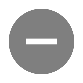 | 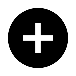 | 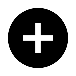 |
| Rajput (2020) | 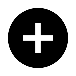 | 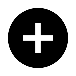 | 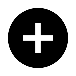 | 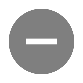 | 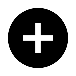 | 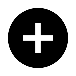 | | 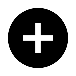 | 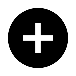 | 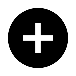 |
| Yeates (2020) | 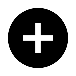 |  |  |  |  |  | |  |  |  |
| Qasim (2020) |  |  |  |  |  |  | |  |  |  |
| Ojetti (2020) |  |  |  |  |  |  | |  |  |  |
| Venter (2020) |  |  |  |  |  |  | |  |  |  |
| Way (2020) |  |  |  |  |  |  | |  |  |  |
| Abdallah (2020) |  |  |  |  |  |  | |  |  |  |
| Chiba (2021) |  |  |  |  |  |  | |  |  |  |
| Walline (2021) |  |  |  |  |  |  | |  |  |  |
| Moyer (2021) |  |  |  |  |  |  | |  |  |  |
| Nia (2021) |  |  |  |  |  |  | |  |  |  |
| Riuttanen (2021) |  |  |  |  |  |  | |  |  |  |
| Kuo (2021) |  |  |  |  |  |  | |  |  |  |
| Rozenfeld (2021) |  |  |  |  |  |  | |  |  |  |
| Hazra (2021) |  |  |  |  |  |  | |  |  |  |
| Matthay (2021) |  |  |  |  |  |  | |  |  |  |
| McGuinness (2021) |  |  |  |  |  |  | |  |  |  |
| Van Aert (2021) |  |  |  |  |  |  | |  |  |  |
| Christey (2021) |  |  |  |  |  |  | |  |  |  |
| Berg (2021) |  |  |  |  |  |  | |  |  |  |
| Devarakonda (2021) |  |  |  |  |  |  | |  |  |  |
| Quraishi (2021) |  |  |  |  |  |  | |  |  |  |
| Kreis (2021) |  |  |  |  |  |  | |  |  |  |
| Ghafil (2021) |  |  |  |  |  |  | |  |  |  |
| Jefferies (2021) |  |  |  |  |  |  | |  |  |  |

*Note: For a study to be considered at a low risk of bias, it was required to have 3 or 4 points in selection, 1 or 2 points in comparability, and 2 or 3 points in outcomes. For a study to be considered at a moderate risk of bias, it was required to have 2 points in selection, 1 or 2 points in comparability, and 2 or 3 points in outcomes. For a study to be considered to be at a high risk of bias, it was required to have 0 or 1 point(s) in selection, or 0 points in comparability, or 0 or 1 point(s) in outcomes.*

Figure A1: % variation of major trauma admissions pre-COVID-19 vs COVID-19 by continent

Figure A2: ISS>12 variation by continent

Figure A3: Patients requiring ICU variation by continent

Figure A4: Mortality of major trauma admitted patients by continent

Figure A5: Road traffic collisions variation of major trauma admitted patients by continent

Figure A6: Assault variation of major trauma admitted patients by continent

Figure A7: Firearm and gunshot variation of major trauma admitted patients by continent

Figure A8: Falls variation of major trauma admitted patients by continent

Figure A9: Self-inflicted suicide attempts variation of major trauma admitted patients by continent

Figure A10: Major trauma variation on the road by continent

Figure A11: Major trauma variation at home by continent

References:

1. Stroup DF, Berlin JA, Morton SC, et al. Meta-analysis of observational studies in epidemiology: a proposal for reporting. Meta-analysis Of Observational Studies in Epidemiology (MOOSE) group. JAMA. 2000;283(15):2008-2012. doi:10.1001/jama.283.15.2008

2. Ritchie H, Mathieu E, Rodés-Guirao L, Appel C, Giattino C, Ortiz-Ospina E, Hasell J, Macdonald B, Beltekian D, Roser M - "Coronavirus Pandemic (COVID-19)". Published online at OurWorldInData.org. Retrieved from: 'https://ourworldindata.org/coronavirus'. 2020.

3. Chhibber-Goel J, Malhotra S, Krishnan NMA, Sharma A. The profiles of first and second SARS-CoV-2 waves in the  top ten COVID-19 affected countries. Journal of Global Health Reports. 2021;5:e2021082. [doi:10.29392/001c.27143](https://doi.org/10.29392/001c.27143)
